# Surgical Reform

**Published:** 1829-04-01

**Authors:** 


### SURGICAL REFORM.

When the last sheet of our last number was actually passing through the press, we saw the account of the meeting, at the Freemason's Tavern, of certain SURGICAL REFORMERS, with the curious string of resolutions then passed. One of the resolutions was moved by a Mr. Hensley, and at the moment of reading the account in the newspapers, we thought we recollected a name somewhat similar over the door of a chemist's shop in the neighbourhood of the Yorkshire Stingo, and we had not time to make the necessary inquiry. By this mistake, we became the unintentional means of drawing odium, or the suspicion of it, on Mr. HENSLEIGH, a most respectable surgeon, residing in Gloucester Place, New Road. This gentleman did not attend the said meeting, and he therefore wrote us a very indignant letter, in contradiction to our statement concerning him, for insertion in our next number. Having learnt from Mr. Craddock that we were in error respecting Mr. Hensleigh, we dispatched a letter to that gentleman apologizing for the allusion to his name, and assuring him that we would correct the mistake through the most public channels. This letter having been received by Mr. Hensleigh, while his own was in transitu to us, changed the whole affair, and convinced him that the error was quite unintentional on our part. The insertion of Mr. Hensleigh's letter then became unnecessary, and we take this opportunity of freeing Mr. H. from all odium attached to the supposed attendance at the above-mentioned meeting, as well as to express our regret at the mode in which the allusion to his name was made in our own columns.

We have been requested by Mr. HENSLEY of Great James Street, Bedford Row, to state that he was not the individual who moved the resolution! In all probability the name was fictitious.

